# Supplementary material for: A true face of Indian married couples: Effect of age and education on control over own sexuality and sexual violence
Source: PLoS One. 2021 Jul 21;16(7):e0254005. doi: 10.1371/journal.pone.0254005 (PMC8294513; doi:10.1371/journal.pone.0254005)
Supplement: Attachment — Submitted filename: comments.docx [file pone.0254005.s001.docx]

The present paper depicts the very important domain of sexuality and sexual violence among Indian couples. The topic fulfils the dearth of couple related picture of sexual violence in India and how age and education of couple is associated with it. However, I have certain comments:

1. Specify total sample in the method section of the abstract.
2. Provide AOR in result section of the abstract.
3. Introduction is well written however a conceptual framework is needed.
4. Please do elaborate the data part and provide citations also.
5. Outcome variable section need a little elaboration
6. Provide some useful explanation and citation for explanatory variables in variable description section.
7. In the result section some changes are needed for example in regression based results provide CI in the result description also e.g., [AOR: 1.90; CI: 1.44-2.90], write the word “significantly more likely or significantly less likely”.
8. Please do check the paper for grammatical mistakes and provide the references in Vancouver style i.e., in big brackets [1], [2-4], [3, 7, 9] etc.

The paper can be accepted after the above minor revisions.
